# The effect of topical tranexamic acid with micro‐needling and micro‐needling alone in treatment of macular amyloidosis

**DOI:** 10.1111/jocd.16517

**Published:** 2024-08-06

**Authors:** Mehdi Gheisari, Fariba Ghalamkarpour, Shima Moslemi Haghighi, Shirin Zaresharifi, Sahar Dadkhahfar, Nabaa Al‐Zubaidi, Leila Ghadirzade Arani, Faeghe Mollaabasi, Reza M. Robati

**Affiliations:** ^1^ Skin Research Center Shahid Beheshti University of Medical Sciences Tehran Iran

**Keywords:** macular amyloidosis, micro‐needling technique, tranexamic acid

## Abstract

**Background:**

Macular amyloidosis is a form of primary localized cutaneous amyloidosis presented by pruritic pigmented macules in rippled or reticulate pattern. The aim of this study was to assess the efficacy of using topical tranexamic acid with micro‐needling comparing to micro‐needling alone in patients with macular amyloidosis.

**Materials and Methods:**

Patients with bilaterally located macular amyloidosis on trunk or upper extremities were recruited in this trial. The skin lesions in all patients were divided into two parts which were randomly assigned to the group of treatment with micro‐needling plus tranexamic acid and the group of micro‐needling alone. There were four sessions of treatment with 2 weeks interval. The percentage of improvement in pigmentation (based on photographs and dermoscopy) and rippling of each group was determined by three blinded dermatologists. The level of patient satisfaction and reduction of pruritus was measured by a questionnaire and defined as a percentage.

**Results:**

Twenty females were enrolled in this study. The mean (SD) patients' age was 39.7 (±10.13) years. Both groups showed improvement in pigmentation based on images, dermoscopy, and rippling pattern. Patients' satisfaction was 46.5% in tranexamic acid group and 47.5% in micro‐needling alone. Nevertheless, there was no significant difference between both groups (*p* value >0.05). Interestingly, the pruritus improved 61.66% after four sessions of treatment in both groups.

**Conclusion:**

Micro‐needling is a suitable modality for decreasing pruritus and pigmentation in macular amyloidosis. However, topical application of tranexamic acid does not lead to additional improvement.

## 1 INTRODUCTION

Macular amyloidosis (MA) is a common form of primary localized cutaneous amyloidosis (PLCA) characterized by pruritic dusky‐brown or grayish pigmented macules in rippled or reticulate pattern. MA is usually symmetrically distributed over the upper back and extensor surface of extremities.^1^ Clavicles, breasts, face, and neck are other sites involved with MA in several reports.^2^ Although the exact etiopathogenesis of macular amyloidosis has remained obscure, the most remarkable risk factors for MA are proved to be race, genetics, female gender, sun exposure, atopy, friction, and autoimmunity.^3^, ^4^ MA is more common among individuals in Asia, South America, and Middle East.^5^ This condition is associated with abnormal deposition of heterogenic amyloid fibrils in extra‐cellular space of papillary dermis and occurs with interaction of genetic and environmental factors.^1^, ^2^ Degeneration of basal keratinocytes plays the major role in pathogenesis of macular amyloidosis.^6^ Since the macular amyloidosis is limited to the skin without any evidence of visceral involvement, treatments of this disease have been centered on the improvement of associated symptoms and cosmetic aspects.^7^

Tranexamic acid or trans‐4‐aminomethyl cyclohexane carboxylic acid (TXA) is a synthetic derivative of lysine commonly used as an antifibrinolytic in the treatment of major bleeding.^8^ TXA blocks the site of lysine on plasminogen and by this way inhibits fibrinolysis.^8^ Moreover, topical administration of TXA prevents the binding of plasminogen to the keratinocytes, and thus prevents the UV‐ induced plasmin activity. These pathways result in a reduction in free arachidonic acid, prostaglandin production, and melanocyte tyrosinase activity.^9^

There are few studies on evaluation of the effect of tranexamic acid on macular amyloidosis.^10^ The aim of this study was to assess the efficacy of using topical tranexamic acid with micro‐needling comparing to micro‐needling alone in patients with macular amyloidosis.

## 2 MATERIALS AND METHODS

This study is a randomized controlled trial conducted in double‐blind manner between March 2021 and March 2022. An informed written consent was obtained from all patients after explanation of the research process.

Patients with more than 18 years of age with the diagnosis of bilateral macular amyloidosis were included in this study. Patients with history of any types of auto‐immune disorders, coagulopathies, and keloidal tendency were excluded from our study. Additionally, those receiving treatment (bleaching agents, laser, dermabrasion, chemical peels) and patients with pregnancy, lactation, and allergy to lidocaine were excluded from the study.

The skin lesions in all patients were divided into two sides which were randomly assigned to the group of treatment with micro‐needling plus tranexamic acid and the group of micro‐needling alone according to the random number allocation method. Detailed history taking and dermatologic examinations were performed by dermatologists. Photographing and dermoscopy of the MA lesions were done at baseline and 2 weeks after the final session using FotoFinder® device.

### 2.1 Treatment protocol

Four sessions of treatment with 2 weeks interval were performed for all patients. In order to prevent any skin contamination, the skin was initially wiped with saline solution. Topical anesthetic cream of 2.5% lidocaine/2.5% prilocaine was applied as a thick coating under occlusion to the treatment areas 30 min before micro‐needling. After removing the anesthetic cream using sterile gas and alcohol‐based disinfectant, skin micro‐needling was performed over both treatment sides with micro‐needling pen that consists of a disposable cartridge of 12 needle tips and adjustable depth of 0.5–1.5 mm. The end point of the procedure was the appearance of erythema and pinpoint bleeding points. Immediately after micro‐needling, 5 cc tranexamic acid (500 mg/5 cc) was applied over the selected side for treatment with tranexamic acid (TXA area). To make our patients blind to the treatment side, 5 cc normal saline was applied on the control side.

The potential side effects of treatment including mild burn, bruise, and acne were monitored and recorded by a dermatologist.

Topical medications were banned until the end of the study. Moreover, sun exposure was avoided by using sunscreens with sun protection factor (SPF) value of more than 50. Before the first session (baseline) and 2 weeks after the end of the treatment, the patients were examined by a dermatologist and photographed with the FotoFinder® device.

### 2.2 Clinical evaluation of lesions

The amount of improvement was measured objectively and subjectively; as the level of patient satisfaction and reduction of pruritus was measured by a questionnaire, and to ensure an unbiased assessment of the treatment outcomes, the comparison of the lesions' photographs was performed using images obtained from photographs and dermoscopy. These images were then independently evaluated by three separate dermatologists, who provided their expert opinions on the degree of improvement observed in each case. The mean results of their assessments were then presented in the Table 1.

**TABLE 1:** Comparison of pigmentation improvement (based on photographs and dermoscopy), rippling improvement, and patient satisfaction between two groups.

Variables	With TA	Without TA	*p*‐value
Pigmentation improvement (based on photographs)	32 ± 13.01	31.62 ± 17.57	0.91
Rippling	24.64 ± 18.34	25.35 ± 18.23	0.72
Pigmentation improvement (based on dermoscopy)	33.75 ± 16.7	37.96 ± 13.07	0.33
Patient satisfaction	46.5 ± 22.24	47.5 ± 22.15	0.16

### 2.3 Statistical analysis

Data was analyzed using SPSS version 16 (SPSS Inc., Chicago, IL). Numerical data were expressed as mean and standard deviation or median and interquartile range (IQR) as appropriate. Qualitative data were expressed as frequency and percentage. Comparison of numerical variables was done using paired *t*‐test for paired (matched) samples when comparing with and without TA values. The correlation between duration and various variables was done using the Spearman rank correlation. *p* values less than 0.05 were considered statistically significant.

## 3 RESULTS

Total of 20 females with the diagnosis of macular amyloidosis were enrolled in this study. The mean (SD) age of our population was 39.7(±10.13) years (range: 18–60 years). The median duration of disease in this study was 7 years (interquartile range3.25, 13.75). The minimum duration of the disease among our population was 2 years and maximum duration was 30 years.

Xerosis and pruritus were the main symptoms among our population occurring in 70% and 68.4%, respectively.

In this study, both treatments (micro‐needling with and without tranexamic acid) were performed on the same patients. Therefore, there was no difference between two groups according to age, sex, duration of disease, and lesional pattern (*p* value>0.05).

**FIGURE 1:**
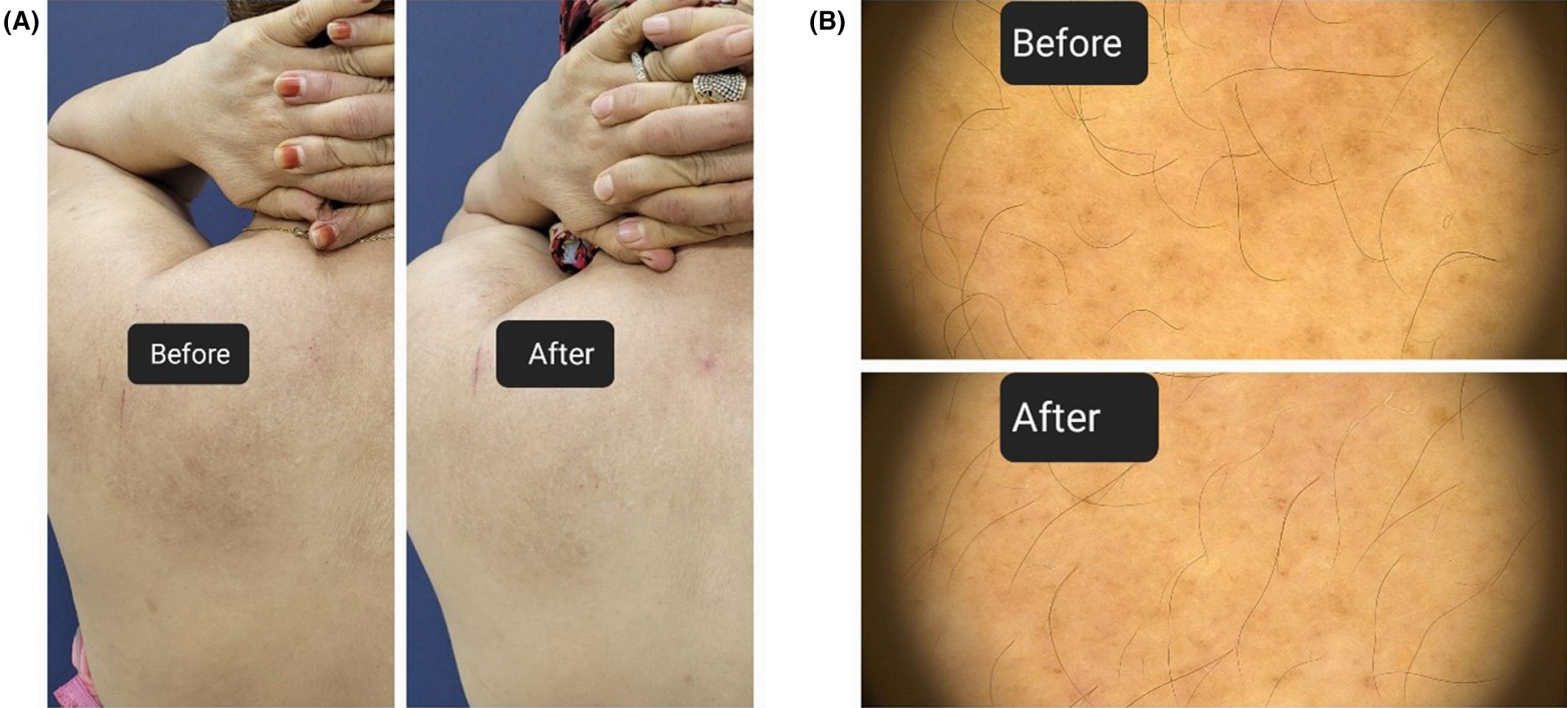
Improvement of lesions after four sessions of treatment. (A) Photograph and (B) dermoscopy.

Paired *t*‐test was used for comparison of the treatment groups. The results of statistical analysis of the improvement in both groups and the comparison of treatments with each other is showed in Table 1 (Figure 1). There was no significant difference between both groups according to the pigmentation improvement based on photographs, dermoscopy, changing in rippling pattern, and patient satisfaction (*p* value>0.05).

Total of 13 patients (68.4%) had pruritus as a common symptom of macular amyloidosis among which near 61.66% improved after four sessions of treatment.

We also assessed the effect of the duration of disease on the improvement factors using Spearman correlation coefficient test which is shown in Table 2.

**TABLE 2:** The association between the duration of disease and the improvement factors.

Duration	Cure with TA	Cure without TA	Rippling with TA	Rippling without TA	Dermoscopy with TA	Dermoscopy without TA	Patient satisfaction with TA	Patient satisfaction without TA	Decrease pruritus with TA
Spearman correlation coefficient	−0.164	0.178	0.589*	0.526*	0.188	0.420	0.082	0.095	−0.276
*p*‐value	0.489	0.453	0.021	0.037	0.485	0.105	0.730	0.690	0.362

* Older lesions showed better responses (improvement of rippling pattern) to treatment.

There was a significant association between duration of the disease and the improvement of rippling pattern in lesions treated with and without tranexamic acid. Older lesions showed better responses to the treatment. (*p* value<0.05).

**FIGURE 2:**
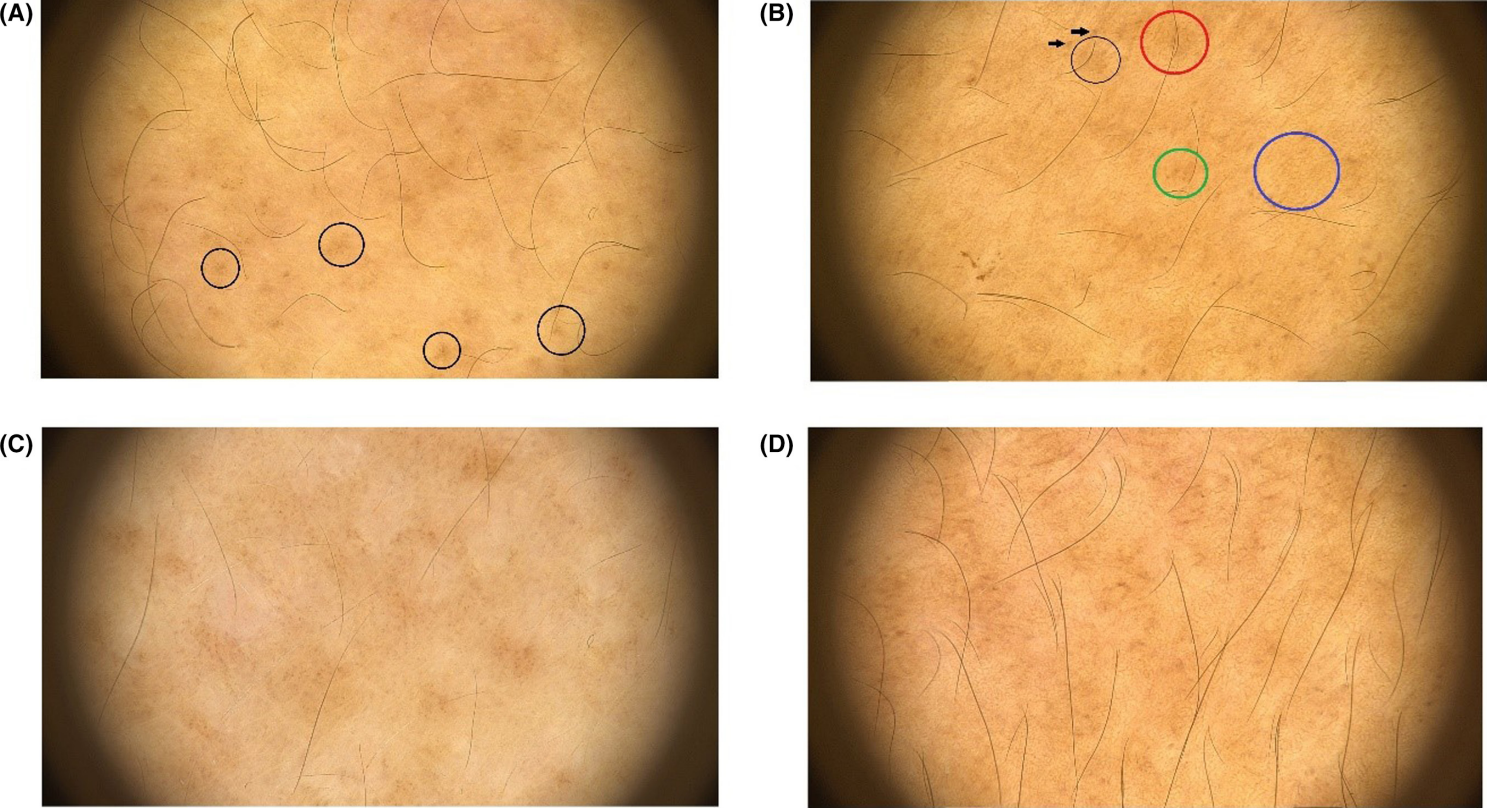
Dermoscopic features of macular amyloidosis. (A) Starburst; (B) Dot discrete(arrows), Dot cluster (green circle), Network (blue circle), Brownish patch (red circle), and Hub and spoke (black circle); (C) Perifollicular rim of hypopigmentation; and (D) Ridge and groove.

As an additional results in our study, we reported the prevalence of dermoscopic features of macular amyloidosis in our population in Table 3. The most common pattern was discrete dots that was seen in 94.4% of our patients. The cluster dots and patchy brownish spots were the next common patterns with prevalence of 83.3% followed by ridge and groove and network patterns which were seen in 55.6% and 38.9% of lesions, respectively (Figure 2).

**TABLE 3:** Macular amyloidosis lesions pattern in dermoscopy.

Lesion pattern	Number	Percentage
Dot (discrete pattern)	17	94.4
Dot (cluster pattern)	15	83.3
Patchy brownish spots	15	83.3
Ridge and groove	10	55.6
Network	7	38.9
Hub and spoke	7	38.9
Perifollicular rim (reduced pigmentation)	6	33.3
Dot (starburst pattern)	1	5.6

In our study, we found that the majority of participants, approximately 70%, did not experience any side effects from the treatment. A small number of participants reported experiencing some mild adverse effects like acne, mild burning sensation, and bruise, which were transient and resolved without additional intervention.

## 4 DISCUSSION

The treatment of macular amyloidosis has always been a major challenge for the dermatologists. Several treatment strategies have been proposed such as topical corticosteroid, DMSO, PUVA (photochemotherapy), UVB phototherapy, Fractional laser, Nd:YAG laser (532 nm and 1064 nm), and Pulsed dye laser treatment. However, none of the therapies has a proper and long‐term effect on the lesions. Tranexamic acid is a synthetic derivative of lysine which can prevent the proliferation of melanocytes and decreases the melanin synthesis in melanocytes leading to the hyperpigmentation improvement. Intralesional TXA has been recently suggested in treatment of macular amyloidosis lesions.^10^

Micro‐needling technique improves transdermal drug delivery by opening micron‐sized channels in the skin. Moreover, it is used as an adjuvant therapy in hyper‐pigmentary diseases such as melasma due to various mechanisms of reduction of hyperpigmentation like increasing the expression of matrix metalloproteinases.^11^

The benefits of intralesional tranexamic acid on pigmentation were assessed more frequently in the treatment of patients with refractory melasma with no serious adverse effects.^12^ Intradermal microinjection of tranexamic acid in melasma lesion in Asian patients evaluated by LEE et al. showed a significant decrease in the MASI score from baseline (13.22 ± 3.02) to 8 and 12 weeks of treatment (9.02 ± 2.62 vs. 7.57 ± 2.54, respectively).

In a relatively similar study in 2018, Saleh et al. evaluated the effect of using topical tranexamic acid with micro‐needling versus micro‐needling alone in the treatment of melasma as a hyperpigmentation disease. That study conducted on 42 patients with melasma randomly divided to 2 groups. Both groups underwent a series of six sessions of micro needling with 2 weeks' interval. The mean melasma area and severity index (MASI) was significantly decreased in both groups. The reduction of MASI score was significantly higher in patients treated with tranexamic acid. That study also evaluated the histopathologic effects of both treatments, which showed a more reduction in epidermal hyperpigmentation and dermal melanophages in patients treated with tranexamic acid than others.^13^

The efficacy of tranexamic acid on treatment of other pigmentary disorders such as infraorbital hyperpigmentation and post‐inflammatory hyperpigmentation (PIH) was also evaluated in some studies. Sirithanabadeekul et al. examined the effect of intradermal tranexamic acid injection on PIH after solar lentigo removal with a Q‐switched.

532‐nm Nd:YAG laser which confirmed the higher reduction in melanin index among patients receiving intradermal tranexamic acid compared with that in controls. Similar to other previous studies, the side effects of tranexamic acid administration were minimal and resolved in the first hour.^14^ In contrast, Kato et al. conducted a similar study on the efficacy of oral tranexamic acid for the prevention of PIH after Q‐Switched 532‐nm Nd:YAG laser for solar lentigines which showed no significant difference in the incidence of PIH, relative melanin value, lightness index, and clinical improvement scores between the two groups of tranexamic acid and control.^15^ The different results of these two studies can be explained by the different route of the tranexamic acid administration. Ghandehari et al. demonstrated that micro‐needling in combination with tranexamic acid leads to a favorable decrease in periorbital hyperpigmentation.^16^

A study conducted by Ostovari et al. evaluated the effectiveness of 532‐nm Q‐switch laser therapy for decreasing pigmentation in macular amyloidosis lesions. Their results showed that 90% of cases achieved a more than 50% reduction in the darkness of patches. In comparison, our findings demonstrated more than 30% reduction of pigmentation.^17^

In a recent study by Ghasemi et al., the effectiveness of two different methods of treating macular amyloidosis was compared.^10^ That study claimed that intralesional injection of tranexamic acid was significantly more effective in improving hyperpigmentation than topical application of Kligmann's combination drug. However, we did not observe additional benefits in the use of topical tranexamic acid after microneedling in our patients. Therefore, based on the mentioned RCTs, in macular amyloidosis the story is different and intradermal tranexamic acid injection led to more significant results than the topical application immediately after microneedling.

To the best of our knowledge, our study is the first study on evaluation of the effect of topical tranexamic acid with micro‐needling and comparison of this method with micro‐needling alone on macular amyloidosis lesions. The results of our study showed no difference between both groups (with and without tranexamic acid) regarding the Pigmentation improvement (based on photographs and dermoscopy), changing rippling pattern, and patient satisfaction. Another finding of this study was the significant association between duration of the disease and improvement of rippling pattern in lesions of both groups. The interesting finding of our study was the significant decrease of pruritus (61.6%) in patients of both groups. However, the exact mechanisms underlying this improvement remain to be fully elucidated. Several potential mechanisms might be at play. Micro‐needling could potentially modulate the release of neurotransmitters and neuropeptides involved in itch sensation or reduce the density of itch‐sensing nerve fibers in the skin. These are speculative explanations, and more research is required to determine the precise mechanisms responsible for the observed reduction in pruritus.

We acknowledge that one limitation of our research is the absence of biopsy data. Since we did not perform biopsies on our patients, we could not directly assess the potential reduction in amyloid deposits and melanophages in their skin. The novelty of our approach, combined with the limited number of previous studies utilizing tranexamic acid or micro‐needling for similar lesions, posed challenges in drawing extensive comparisons with existing research.

## 5 CONCLUSION

Micro‐needling is a suitable modality for decreasing pruritus and pigmentation in macular amyloidosis. However, topical application of tranexamic acid does not lead to additional improvement. Due to the acceptable effects of this drug in improving other hyper pigmentary diseases such as melasma without any major side effects, it is necessary to conduct future studies on evaluation of the exact effects of administering tranexamic acid on macular amyloidosis using various routes, such as oral, topical, intradermal injections, and with micro‐needling technique. On another hand, according to the significant decrease of pruritis as an irritating symptom of macular amyloidosis in lesions treated using micro‐needling alone, this effective, inexpensive, and safe treatment strategy could be considered as adjunctive therapy for such patients. However, due to the limitations of studies in this regard and its unknown mechanism, this issue should be investigated in future studies. Furthermore, future studies can evaluate effect of micro‐needling on some chronic bothersome diseases like lichen simplex chronicus.

## AUTHOR CONTRIBUTIONS

All authors contributed to the study conception and design. All authors read and approved the final manuscript. *Conceptualization*: Mehdi Gheisari, Fariba Ghalamkarpour. *Methodology*: Mehdi Gheisari, Reza M Robati. *Writing—original draft preparation*: Shima Moslemi Haghighi. Writing‐review and editing: Shirin Zare Sharifi, Sahar Dadkhahfar, Reza M Robati. *Data gathering and analysis*: Shima Moslemi Haghighi, Leila Ghadirzade, Faeghe Mollaabasi, Nabaa Al‐Zubaidi. *Supervision*: Mehdi Gheisari.

## CONFLICT OF INTEREST STATEMENT

The authors declare that they have no conflict of interest.

## ETHICS APPROVAL

Scientific procedures were done according to clinical Ethic Committee at Shahid Beheshti University of Medical Sciences, Tehran, Iran.

## Data Availability

The data that support the findings of this study are available from the corresponding author upon reasonable request.

## References

[1] Sonthalia S , Agrawal M , Sehgal V . Dermoscopy of macular amyloidosis. Indian Dermatol Online J. 2021;12(1):203‐205. 33768060 10.4103/idoj.IDOJ_507_19 PMC7982011

[2] Kombettu AP , Gurumurthy C , Rangappa V , Shastry V . Frictional melanosis and macular amyloidosis–exploring the link. Pigment International. 2022;9(3):166‐175.

[3] Hamie L , Haddad I , Nasser N , Kurban M , Abbas O . Primary localized cutaneous amyloidosis of keratinocyte origin: an update with emphasis on atypical clinical variants. Am J Clin Dermatol. 2021;22:667‐680. 34286474 10.1007/s40257-021-00620-9

[4] Nilforoushzadeh MA , Zolghadr S , Heidari‐Kharaji M , Alavi S , Mahmoudbeyk M . A comparative study of the efficacy of fractional neodymium‐doped yttrium aluminum garnet (Nd: YAG) laser therapy alone and in combination with erbium: YAG laser therapy: tracing and objective measurement of melanin index in macular amyloidosis. Lasers Med Sci. 2020;35:1171‐1177. 31916020 10.1007/s10103-020-02954-y

[5] Aslani FS , Kargar H , Safaei A , Jowkar F , Hosseini M , Sepaskhah M . Comparison of immunostaining with hematoxylin‐eosin and special stains in the diagnosis of cutaneous macular amyloidosis. Cureus. 2020;12(4):e7606. 32399340 10.7759/cureus.7606 PMC7213674

[6] Liu H , Qiu B , Yang H , et al. AHNAK, regulated by the OSM/OSMR signaling, involved in the development of primary localized cutaneous amyloidosis. J Dermatol Sci. 2023;110(2):53‐60. 37100691 10.1016/j.jdermsci.2023.04.004

[7] Weidner T , Illing T , Elsner P . Primary localized cutaneous amyloidosis: a systematic treatment review. Am J Clin Dermatol. 2017;18:629‐642. 28342017 10.1007/s40257-017-0278-9

[8] Colferai MMT , Miquelin GM , Steiner D . Evaluation of oral tranexamic acid in the treatment of melasma. J Cosmet Dermatol. 2019;18(5):1495‐1501. 30536592 10.1111/jocd.12830

[9] Maeda K . Mechanism of action of topical tranexamic acid in the treatment of melasma and sun‐induced skin hyperpigmentation. Cosmetics. 2022;9(5):108.

[10] Ghassemi M , Roohaninasab M , Kamani SA , Sadeghzadeh‐Bazargan A , Goodarzi A . Comparison of the efficacy and safety of intralesional injection of tranexamic acid and the topical application of Kligman combination drug in the treatment of macular amyloidosis. Dermatol Ther. 2022;35(1):e15213. 34797597 10.1111/dth.15213

[11] Singh A , Yadav S . Microneedling: advances and widening horizons. Indian Dermatol Online J. 2016;7(4):244‐254. 27559496 10.4103/2229-5178.185468 PMC4976400

[12] Lueangarun S , Sirithanabadeekul P , Wongwicharn P , et al. Intradermal tranexamic acid injection for the treatment of melasma: a pilot study with 48‐week follow‐up. J Clin Aesthet Dermatol. 2020;13(8):36‐39. PMC7595366 33178380

[13] Saleh FY , Abdel‐Azim ES , Ragaie MH , Guendy MG . Topical tranexamic acid with microneedling versus microneedling alone in treatment of melasma: clinical, histopathologic, and immunohistochemical study. J Egypt. 2019;16(2):89‐96.

[14] Sirithanabadeekul P , Srieakpanit R . Intradermal tranexamic acid injections to prevent post‐inflammatory hyperpigmentation after solar lentigo removal with a Q‐switched 532‐nm Nd: YAG laser. J Cosmet Laser Ther. 2018;20(7–8):398‐404. 29505310 10.1080/14764172.2018.1444770

[15] Kato H , Araki J , Eto H , et al. A prospective randomized controlled study of oral tranexamic acid for preventing postinflammatory hyperpigmentation after Q‐switched ruby laser. Dermatologic Surg. 2011;37(5):605‐610. 10.1111/j.1524-4725.2011.01957.x 21457392

[16] Ghandehari R , Robati RM , Niknezhad N , Hajizadeh N , Tehranchinia Z . Efficacy and safety of fractional CO2 laser and tranexamic acid versus microneedling and tranexamic acid in the treatment of infraorbital hyperpigmentation. J Dermatolog Treat. 2022;33(3):1391‐1396. 32893707 10.1080/09546634.2020.1819527

[17] Ostovari N , Mohtasham N , Oadras MS , Malekzad F . 532‐nm and 1064‐nm Q‐switched Nd: YAG laser therapy for reduction of pigmentation in macular amyloidosis patches. J Eur Acad Dermatol Venereol. 2008;22(4):442‐446. 18363913 10.1111/j.1468-3083.2007.02473.x

